# Case report: Multimodal imaging diagnosis of a giant coronary artery fistula: A report of two cases

**DOI:** 10.3389/fcvm.2022.986078

**Published:** 2022-10-26

**Authors:** Mohammadbagher Sharifkazemi, Reza Mohseni-Badalabadi, Ali Hosseinsabet, Alimohammad Hajizeinali

**Affiliations:** ^1^Department of Cardiology, Nemazee Hospital, Shiraz University of Medical Sciences, Shiraz, Iran; ^2^Department of Cardiology, Tehran Heart Center, Tehran University of Medical Sciences, Tehran, Iran; ^3^Department of Interventional Cardiology, Tehran Heart Center, Tehran University of Medical Sciences, Tehran, Iran

**Keywords:** coronary vessels, coronary artery disease, multimodal imaging, fistula, vascular fistula

## Abstract

Being a very rare cardiac disease, most cases of coronary artery fistula (CAF) are genetic. Complications such as coronary steal syndrome, myocardial infarction, heart failure, or tamponade can manifest following the abnormal communication that the fistula creates between the coronary arteries and cardiac chambers or major vessels and the subsequent shunt. Most CAFs are small and asymptomatic, making diagnosis difficult. In symptomatic patients, the initial diagnostic workup is generally made with chest radiography and electrocardiography. Other imaging modalities have also been suggested to improve diagnostic accuracy. Cardiac catheterization and coronary angiography are currently the gold standard for diagnosis and planning the intervention, as they can recognize the quantum of the shunt as well as complications of a fistulous track (e.g., aneurysm formation, thrombus, leak, and the number of openings to the receiving chamber/vessel); however, this invasive method may be associated with risk. Herein, we report two patients with giant CAFs, one from the left circumflex artery to the coronary sinus and the other to the superior vena cava. Moreover, we describe how multimodal imaging, including two- and three-dimensional transesophageal echocardiography, coronary cineangiography, coronary computed tomography angiography, and enhanced chest computed tomography, can facilitate diagnosis and estimate the disease course in such patients. We believe that using multimodal imaging cannot only help the initial diagnosis regarding the presence of a CAF and the accurate anatomical site of the fistula in the patient but can also help predict the disease course and choose the most suitable treatment modality. Therefore, we suggest multimodal imaging be done to diagnose patients suspected of CAF. However, invasive cineangiography should be necessarily followed, regardless of whether an intervention is planned or not.

## Introduction

Coronary artery fistula (CAF) is among the rare cardiac diseases comprising about 0.002% of the general population and 0.4% of all cardiac malformations (1). It is mainly a congenital heart disease but can also be acquired from trauma, infection, or as a result of an iatrogenic injury such as intracardiac operations, transcutaneous myocardial biopsy, and coronary angioplasty (2).

Coronary artery fistula induces an abnormal communication between the coronary arteries and cardiac chambers or major vessels of systemic or pulmonary circulation without an interposed capillary bed. Consequently, blood is drawn away from the normal cardiac circulation (known as coronary “steal” syndrome), widening pulse pressure (1, 3). About 60% of fistulas arise from the right coronary artery, and 90% drain to the right chamber (4, 5). A large amount of shunt drainage into the venous side of the systemic circulation increases blood volume in the right heart structures and induces pulmonary arterial hypertension (PAH); untreated cases may develop right-sided heart failure or cardiac tamponade as a result of rupture or thrombosis of fistula or arterial aneurysm (1, 3, 4). However, when the fistula drains to the left atrium or left ventricle, the left heart volume is only increased, producing run-off from the aorta, resulting in no shunt (1, 3, 4).

Despite being described nearly two centuries ago in 1841, the real incidence and clinical course of CAF have not been well-defined since more than half of the cases have a small fistula and are accordingly asymptomatic and clinically undetectable. Patients with large CAFs may present with an unexplained systolic-diastolic heart murmur (with a crescendo-decrescendo pattern) or conduction abnormalities, atrial fibrillation, and ventricular tachyarrhythmia on physical examination. CAF-associated complications such as myocardial infarction or chronic myocardial ischemia can be detected on an electrocardiogram (ECG) and by volume overload on a chest radiograph; however, these modalities are insufficient for accurate diagnosis of CAF itself (4). More accurate imaging modalities, such as two- and three-dimensional transthoracic and transesophageal echocardiograms (2D and 3D TTE and TEE), Doppler echocardiography, enhanced chest computed tomography (CT), and coronary computed tomography angiography (CCTA), aid and facilitate diagnosis of the fistula site, origin, and terminus (4). In almost all intervention centers, 2D and 3D TEE and TTE are used in the supplement to cineangiography for verifying the exact anatomy of this anomaly and successful CAF closure (6), which further shows the supplementary role of modern imaging techniques. Herein, we describe and compare the disease course of two patients with CAFs, left circumflex artery (LCx), one to the coronary sinus and the other LCx to superior vena cava, to emphasize the role of multimodal imaging in the diagnosis and prognosis in patients suspected of CAF.

## Case presentation

### Case#1: Giant coronary artery fistula between proximal left circumflex artery and coronary sinus

A 43-years-old woman had a long history of mitral valve (MV) prolapse and tricuspid regurgitation, for which she had undergone MV replacement with a bioprosthetic MV (mosaic #31) plus tricuspid valve repair 12 years ago. Two years after her operation, the results of routine follow-up echocardiography and 2D TEE raised the possibility of a connection between the left circumflex artery and coronary sinus (Supplementary Figure 1), confirmed by more investigations such as conventional coronary angiography and CCTA. At that time, the patient refused cardiac surgery and missed her follow-up appointments for a long period.

She was referred to our center by the cardiologist echocardiographer for doing a transesophageal echocardiographic evaluation of valve function to rule out malfunction of the bioprosthetic MV. Her chief complaint was progressive shortness of breath, and 2D TTE examination revealed an excess transmitral gradient (peak gradient of 24 mmHg and mean gradient of 11 mmHg) accompanied by severe PAH (systolic pulmonary artery pressure = 65 mmHg) with an insignificant right to left shunt.

**FIGURE 1:**
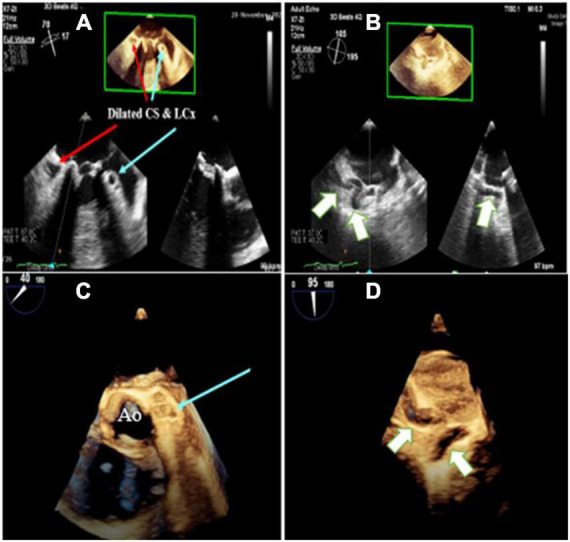
Three-dimensional zoom mid esophageal echocardiography **(A)** shows dilated left circumflex artery (LCX; light blue arrow) and coronary sinus (CS), **(B)** tortuous, giant coronary fistula (white arrow), **(C,D)** mid esophageal full volume illustrating the same findings.

**FIGURE 2:**
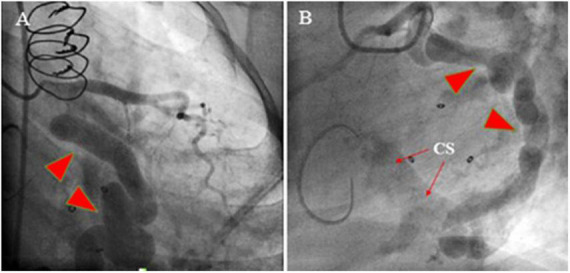
Coronary artery cineangiography in 30° RAO (left; **A**) and 60° LAO (right; **B**) projections show a giant coronary artery (red isosceles triangles) starting from the proximal left circumflex artery and terminating in the same artery (narrow arrows).

**FIGURE 3:**
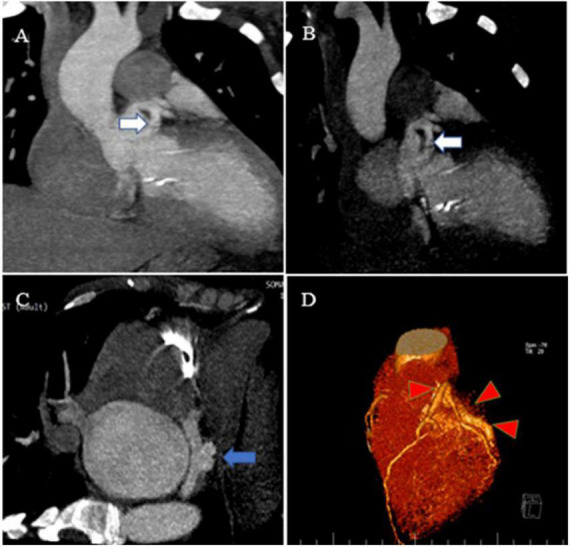
Contrast-enhanced CT scan of the chest, as well as CCTA, show the proximal site of a giant fistula originating from the left circumflex artery (white arrow), draining into the coronary sinus and forming a pouch (blue arrow). Reconstruction of coronaries illustrates a giant fistula (red color arrowheads).

In the recent referral of the patient, 3D TEE was performed, which clearly identified CAF courses and low-velocity flow throughout the whole cardiac cycle, which drained into the coronary sinus (venous system) (Figure 1). Moreover, coronary cineangiography (Figure 2) and contrast-enhanced CT scan of the chest, as well as CCTA (Figure 3), demonstrated the anatomical course of a giant CAF clearly. Therefore, she was referred to an interventionist, but the patient refused further therapeutic intervention once again. As the patient was not evaluated using angiography before surgery, we cannot be sure about the cause, and it might have been iatrogenic.

### Case#2: Giant coronary artery fistula between proximal left circumflex artery and superior vena cava

**FIGURE 4:**
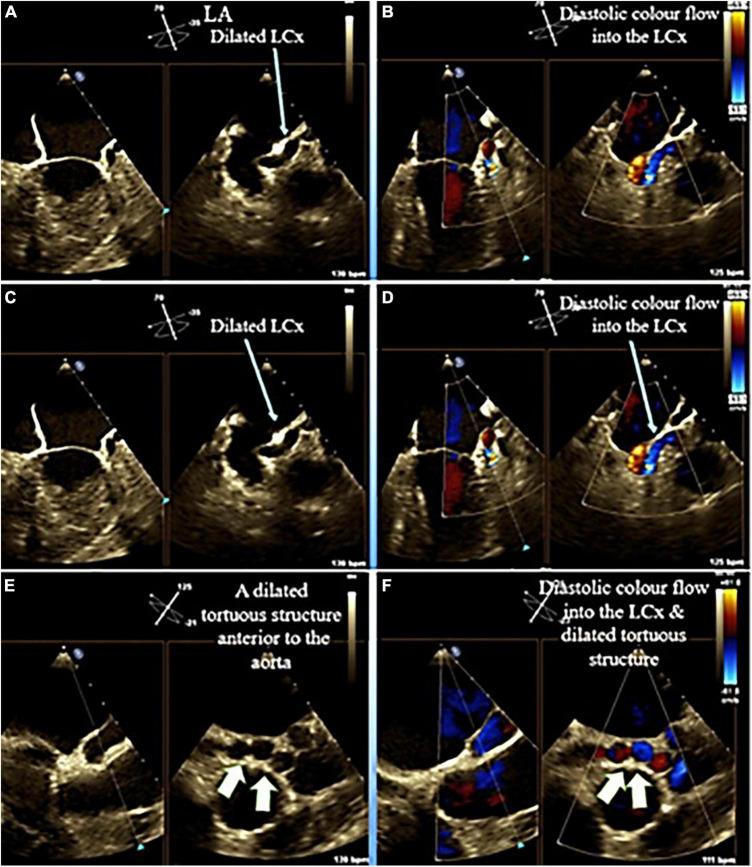
Mid esophageal echocardiography in different views illustrates dilated left circumflex (light blue arrow) as well as a giant tortuous structure (fistula) (white arrow) posterior to the aorta.

**FIGURE 5:**
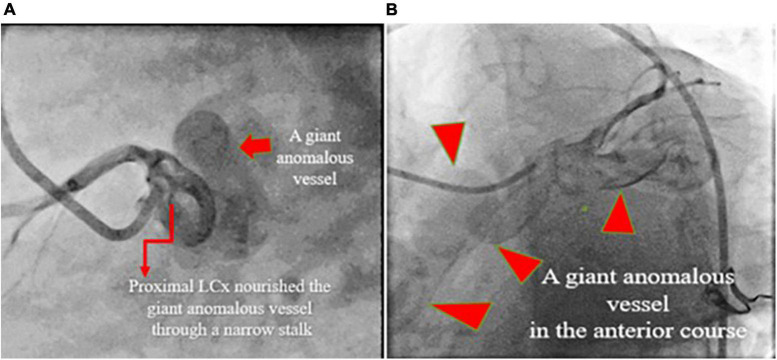
Coronary artery cineangiography in 70° LAO (left; **A**) and 30° LAO (right; **B**) projections show a giant coronary fistula (red isosceles triangles) starting from the proximal left circumflex artery.

**FIGURE 6:**
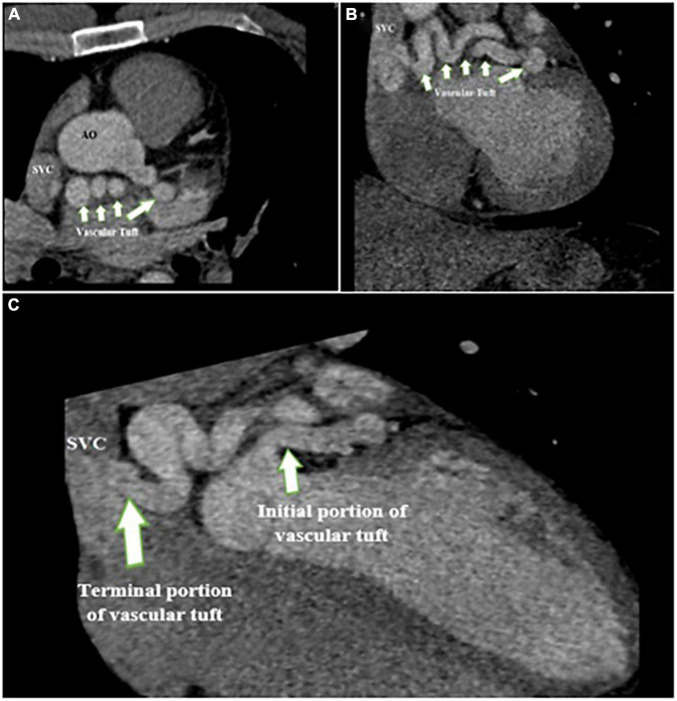
Contrast-enhanced CT scan of the chest shows the proximal site of giant fistula (white arrow) originating from the left circumflex artery with drainage into the superior vena cava.

A 28-year-old man with a history of frequent bouts of paroxysmal atrial fibrillation for several months was scheduled for elective electrical cardioversion and referred to our echocardiography center for TEE on the same day of cardioversion. He had no other remarkable history. TTE results showed the top normal size of both atria and left ventricle, normal biventricular systolic function with no PAH or significant left to right shunt (Qp/Qs = 1.2) (Figure 4). TEE (2D and 3D) showed CAF courses and low-velocity flow throughout the whole cardiac cycle, which drained into the superior vena cava (Supplementary Figure 2). Moreover, coronary cineangiography (Figure 5) and enhanced chest CT (Figure 6) clearly demonstrated the anatomical course of a giant CAF. He underwent cardioversion, and the rhythm resumed to normal sinus rhythm; however, he preferred to leave the hospital and postpone CAF therapeutic intervention.

Patients gave consent for all steps of the diagnostic procedure and cooperated with the physician. The heart team was open to listening to patients’ opinions and reflected them in the medical records, but, both patients decided to postpone the suggested treatments.

## Discussion

In these two case reports, we described how multimodal imaging, including 2D and 3D TEE, coronary cineangiography, and contrast-enhanced CT scan of the chest as well as CCTA, could verify the exact anatomy of CAF, as documented in the provided figures. The first case was suspected of CAF using 2D TEE 10 years ago, 2 years after valvular replacement surgery, but she did not follow her problem until she became symptomatic (progressive shortness of breath). Therefore, her symptoms were suspected to be related to malfunction of the bioprosthetic MV, which was likely to undergo degenerative changes and become stenotic (considering the transmitral gradient of peak 24 and mean 11 at rest with severe PAH); in other words, we assume that CAF had a small or even no role in her symptoms. The second case was scheduled to be managed by electrical cardioversion for his paroxysmal atrial fibrillation, which was found to be related to his CAF using multimodal imaging.

These two patients did not have two of the most common symptoms reported for CAF, namely chest pain and murmur. Also, shortness of breath, the third common symptom, was only observed in one case #1 (7). Furthermore, the types of CAF were not the common ones previously reported, such as coronary to the pulmonary artery or cardiac chamber (8). The clinical course of these two patients depicts the rarity of CAF. Physicians and cardiologists may easily miss this significant condition considering the diversity of its clinical symptoms. In our patients, the involved coronary artery and the drainage site of the fistula were as common as those previously reported. Among 56 patients diagnosed with CAF by CCTA, only one had coronary to superior vena cava fistula (8), and among 29 patients with LCX CFA, the site of fistula drainage was coronary sinus in only one patient (9). Another distinctive feature of the two cases presented here was the large size of the CAF, which has rarely been reported previously (10).

The clinical course of these two patients necessitates careful and close examinations using more accurate diagnostic methods alongside routine methods such as ECG and TTE. Here, both cases were diagnosed as CAF only after being examined with TEE. TTE and TEE play a key role in depicting CAF anatomy and hemodynamic changes with the advantage of lacking ionizing radiation or contrast material (4). Some studies have ascertained that CCTA was the modality of choice for CAF diagnosis (11, 12).

For multimodal imaging, we used coronary cineangiography, and contrast-enhanced CT scan of the chest as well as CCTA. Besides being able to diagnose CAF, these images could also verify the size of the fistula and disease course (to the right or left heart and resulting shunt). Performing pre-treatment coronary angiography has also been suggested in such patients since it can determine more accurate details of the patients’ conditions, including blood flow patterns, device landing zones, and surgical ligation sites, which may not be fully evident on CT or magnetic resonance images (13); however, it imposes a greater risk because of its invasiveness, compared with CCTA. In a large-scale study on 12,457 patients undergoing coronary angiography, 112 patients had coronary anomalies, among whom only 10.7% were found to have CAF; therefore, the overall prevalence of CAF was 0.096% (14). Similarly, in another study, CAF was found in 0.08% of 11,350 patients who underwent coronary angiography (15). Improved imaging techniques following the development of modern technology may also have influenced the more accurate frequencies reported. Other imaging methods, such as intravascular ultrasound and optical coherence tomography, have also been suggested for the diagnosis and treatment of plaque vulnerability (16), coronary artery aneurysm (17), and dissection (18); however, the diagnostic accuracy of these imaging methods has not been established in CAF, and more studies are required in this regard.

Incorporating these multimodal imagings when dealing with patients with CAF enables physicians to benefit from the advantages of each, which also facilitates complete planning of the treatment strategy and may also help reduce long-term adverse outcomes (19). However, currently, physicians select an imaging modality compatible with their own clinical experience, as there are no specific guidelines determining the gold standard imaging modality for CAF detection. Further studies are also required to determine the effect of imaging modalities on patients’ outcomes.

The mainstay of CAF management includes surgical closure versus percutaneous transcatheter closure (TCC) (20). Since CAFs are rare, and there are few studies on the patients’ outcomes and suitability of treatments, it is difficult to determine the most appropriate management. CAF occlusion in 33 patients using TCC (with the placement of coils in 31, detachable balloons in 10, umbrella devices in 2, covered stent in 1, and a combination of detachable balloons and coils in 2) was successful in 83% of the patients and complete closure in 91% at follow-up (21). Although TCC has been suggested since 1982, patients may require re-catheterization or may develop complications, such as transient ischemic changes on ECG and unretrieved device embolization, which may result in death (21). Surgery is mainly used in cases requiring cardiopulmonary bypass for correction of additional cardiac lesions, including patent ductus arteriosus, atrial septal defect, mitral/aortic regurgitation, RCA atresia (i.e., a rare congenital malformation characterized by the absence of right coronary ostium), and coarctation (21). The closure is rigorously suggested in pediatric patients with congenital CAFs, and TCC is suggested as an effective treatment method with fewer complications (19, 22). Studies on the adult population have also reported that TCC was a safe and effective method for the treatment of CAFs (23, 24). In our study, both cases refused the suggested treatment; therefore, we cannot discuss the outcome of treatment in these patients. Further studies are required to determine the long-term outcome of CAF treatment.

## Conclusion

Here, we reported two cases of giant LCX CAF, who were not diagnosed during routine examinations by the cardiologist and were referred for echocardiographic examination with other clinical suspicions. The use of multimodality imaging for these two patients was found as an effective tool for verifying the size of the fistula, the exact anatomic site, source and drainage site, and the resulting shunt. Therefore, we anticipate that using multimodal imaging is beneficial in initial diagnosis, accurate anatomical site of fistula, and prediction of disease course. Therefore, we suggest multimodal imaging be performed in patients suspected of CAF.

## Data availability statement

The raw data supporting the conclusions of this article will be made available by the authors, without undue reservation.

## Ethics statement

Ethical review and approval was not required for the study on human participants in accordance with the local legislation and institutional requirements. The patients/participants provided their written informed consent to participate in this study. Written informed consent was obtained from the individual(s) for the publication of any potentially identifiable images or data included in this article.

## Author contributions

MS and RM-B performed echocardiographies and reviewed coronary angiographies and CCTAs. AHO performed echocardiographies. AHA performed coronary angiographies. MS condensed and collated visualizations for this manuscript. All authors have made substantial contributions to treatment planning, provided the literature review, drafted the manuscript, revised it critically, and gave final approval for publishing.

## Supplementary material

Supplementary Figure 1 The parasternal long-axis view in TTE shows **(A)** dilated coronary sinus (white arrow), **(B)** a well-seated bioprosthetic mitral valve in AC4 view, **(C)** mid esophageal view in two-dimensional transesophageal echocardiography, showing a tortuous, giant vascular tuft; coronary artery fistula (white arrow), and **(D)** blue and red colors in color Doppler study indicates “to and fro” movement of the vascular tuft regarding the probe.

Supplementary Figure 2 Mid esophageal full volume illustrating dilated left circumflex (light blue arrow).
