# Supplementary figures and images for: Case report: Multimodal imaging diagnosis of a giant coronary artery fistula: A report of two cases
Source: Front Cardiovasc Med. 2022 Oct 26;9:986078. doi: 10.3389/fcvm.2022.986078 (PMC9644096; doi:10.3389/fcvm.2022.986078)

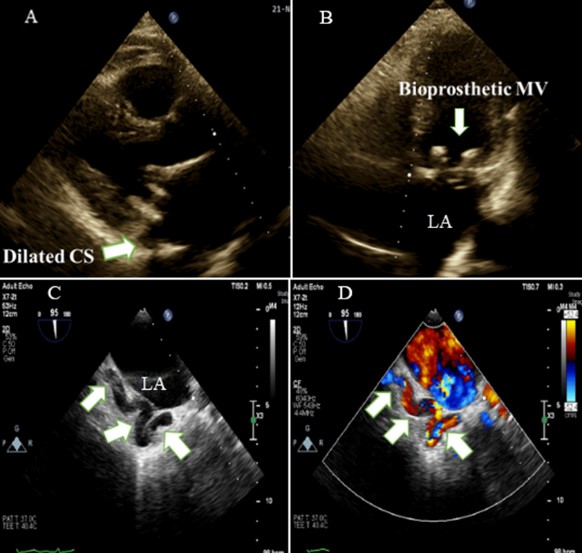

Supplement: Supplementary Figure 1 — The parasternal long-axis view in TTE shows (A) dilated coronary sinus (white arrow), (B) a well-seated bioprosthetic mitral valve in AC4 view, (C) mid esophageal view in two-dimensional transesophageal echocardiography, showing a tortuous, giant vascular tuft; coronary artery fistula (white arrow), and (D) blue and red colors in color Doppler study indicates “to and fro” movement of the vascular tuft regarding the probe. [file Image_1.JPEG]

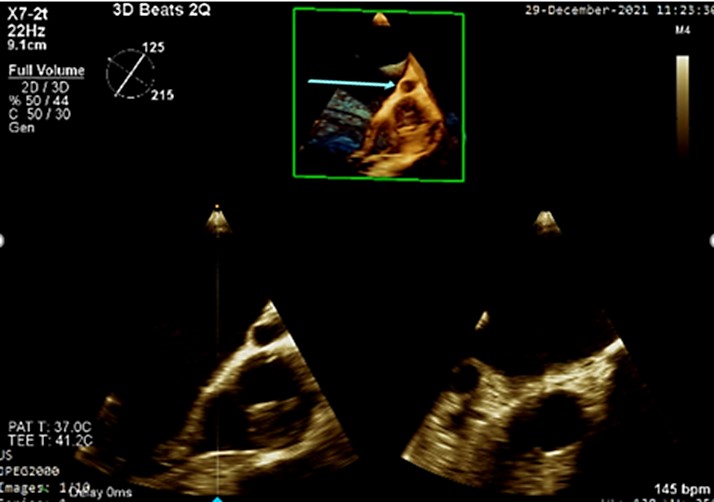

Supplement: Supplementary Figure 2 — Mid esophageal full volume illustrating dilated left circumflex (light blue arrow). [file Image_2.JPEG]
